# Serum microRNA array analysis identifies miR-140-3p, miR-33b-3p and miR-671-3p as potential osteoarthritis biomarkers involved in metabolic processes

**DOI:** 10.1186/s13148-017-0428-1

**Published:** 2017-12-12

**Authors:** E. Ntoumou, M. Tzetis, M. Braoudaki, G. Lambrou, M. Poulou, K. Malizos, N. Stefanou, L. Anastasopoulou, A. Tsezou

**Affiliations:** 1 Laboratory of Cytogenetics and Molecular Genetics, Faculty of Medicine, Biopolis, University of Thessaly, 41500 Larissa, Greece; 2 Laboratory of Medical Genetics, Medical School, National and Kapodistrian University of Athens, Athens, Greece; 3 First Department of Pediatrics, Medical School, National and Kapodistrian University of Athens,, Athens, Greece; 4 University Research Institute for the Study and Treatment of Childhood Disease and Malignant Diseases, National and Kapodistrian University of Athens, “Aghia Sophia” Children’s Hospital, Athens, Greece; 5 Department of Orthopaedics, Faculty of Medicine, University of Thessaly, Larissa, Greece; 6 Department of Biology, Faculty of Medicine, University of Thessaly, Larissa, Greece

**Keywords:** Biomarker, Circulating miRNAs, Hsa-miR-140-3p, Hsa-miR-33b-3p, Hsa-miR- 671-3p, Metabolic, miR-array, Osteoarthritis

## Abstract

**Background:**

MicroRNAs (miRNAs) in circulation have emerged as promising biomarkers. In this study, we aimed to identify a circulating miRNA signature for osteoarthritis (OA) patients and in combination with bioinformatics analysis to evaluate the utility of selected differentially expressed miRNAs in the serum as potential OA biomarkers.

**Methods:**

Serum samples were collected from 12 primary OA patients, and 12 healthy individuals were screened using the Agilent Human miRNA Microarray platform interrogating 2549 miRNAs. Receiver Operating Characteristic (ROC) curves were constructed to evaluate the diagnostic performance of the deregulated miRNAs. Expression levels of selected miRNAs were validated by quantitative real-time PCR (qRT-PCR) in all serum and in articular cartilage samples from OA patients (*n* = 12) and healthy individuals (*n* = 7). Bioinformatics analysis was used to investigate the involved pathways and target genes for the above miRNAs.

**Results:**

We identified 279 differentially expressed miRNAs in the serum of OA patients compared to controls. Two hundred and five miRNAs (73.5%) were upregulated and 74 (26.5%) downregulated. ROC analysis revealed that 77 miRNAs had area under the curve (AUC) > 0.8 and *p* < 0.05. Bioinformatics analysis in the 77 miRNAs revealed that their target genes were involved in multiple signaling pathways associated with OA, among which FoxO, mTOR, Wnt, pI3K/akt, TGF-β signaling pathways, ECM-receptor interaction, and fatty acid biosynthesis. qRT-PCR validation in seven selected out of the 77 miRNAs revealed 3 significantly downregulated miRNAs (hsa-miR-33b-3p, hsa-miR-671-3p, and hsa-miR-140-3p) in the serum of OA patients, which were in silico predicted to be enriched in pathways involved in metabolic processes. Target-gene analysis of hsa-miR-140-3p, hsa-miR-33b-3p, and hsa-miR-671-3p revealed that *InsR* and *IGFR1* were common targets of all three miRNAs, highlighting their involvement in regulation of metabolic processes that contribute to OA pathology. Hsa-miR-140-3p and hsa-miR-671-3p expression levels were consistently downregulated in articular cartilage of OA patients compared to healthy individuals.

**Conclusions:**

A serum miRNA signature was established for the first time using high density resolution miR-arrays in OA patients. We identified a three-miRNA signature, hsa-miR-140-3p, hsa-miR-671-3p, and hsa-miR-33b-3p, in the serum of OA patients, predicted to regulate metabolic processes, which could serve as a potential biomarker for the evaluation of OA risk and progression.

**Electronic supplementary material:**

The online version of this article (10.1186/s13148-017-0428-1) contains supplementary material, which is available to authorized users.

## Background

Osteoarthritis (OA) is the most common chronic degenerative joint disease and a leading cause of pain and disability. Its prevalence is steadily increasing and is expected to be the greatest cause of disability by 2030 [1]*.* OA is defined as a heterogeneous disease consisting of a group of distinct joint disorders with common biological, morphological, and clinical outcomes [2]. To date, several risk factors, including aging, obesity, sex, joint injury, and heredity have been implicated in disease development and progression [3, 4]. Recently, an association has been suggested between OA and metabolic syndrome or metabolic risk factors, as hypertension, overweight, dyslipidemia, and insulin resistance, and a new OA subtype, metabolic OA, has been introduced [5–7]. As the above metabolic factors can be modified, it becomes evident that understanding the molecular mechanisms which are involved in OA pathogenesis is essential for the discovery of OA personalized therapies. However, until now, despite the significant burden that OA imposes in patients and in healthcare systems [8], there is absence of effective non-arthroplasty treatment options for disease management. One major challenge for the development of effective therapy for OA is the detection of the disease at an early stage. To date, the standard method for disease diagnosis and severity is the radiograph, which appears to be limited to detection in late disease stages and has weakness in monitoring disease progression [9]. Therefore, the identification of non-invasive and sensitive serum biomarkers will assist OA diagnosis, prognosis, and early treatment before the onset of radiographic findings.

MicroRNAs (miRNAs) are a novel group of universally present small non-coding RNAs, approximately 22–28 nucleotides in length, which regulate gene expression at the post-transcriptional level through mRNA degradation or translational repression. Each miRNA can regulate a large number of genes by binding mainly to the 3′UTR of mRNA targets [10]. Recent studies have implicated miRNAs as promising clinical biomarkers in various diseases including cancer, diabetes, and cardiovascular disease [11–14]. MiRNAs have also been associated with processes involved in OA pathogenesis, such as cartilage homeostasis, extracellular matrix regulation, endochondral ossification, bone metabolism, apoptosis, autophagy, inflammation, and lipid metabolism [15, 16]. Our group was among the pioneers to suggest the involvement of miRNAs in OA pathogenesis by demonstrating the differential expression of 16 miRNAs in OA compared to healthy articular cartilage [17].

Recently, the use of microarrays for the global characterization of miRNA expression profiling is becoming a strong research tool that provides useful information on the pathogenesis of many diseases [12]. Several microRNA profiling studies have been performed in different OA tissues and have led to the identification of numerous differentially expressed miRNAs between OA joint tissues and controls [17, 18]. However, in the majority of these studies, the number of miRNAs tested was limited, ranging from 157 to 723 miRNAs, compared to 2588 mature miRs recently released from the Sanger Institute and University of Manchester miRbase (release June 2014) allowing for many more miRNAs to be identified [19].

Moreover, miRNAs have been discovered in circulation, however, the origin and the biological functions of the circulating miRNAs in a disease remain poorly understood. Evidence suggests that circulating miRNAs could be transferred in the circulation through microvesicles, exosomes, Ago protein complexes, or HDL and that they are either byproducts of the cellular content or are mediating the inter-cellular signaling reflecting the pathophysiological state of the cell [20–23]. Alterations in their expression levels in abnormal conditions along with their stability in circulation and surprisingly long half-life of approximately 5 days in plasma [24] have implicated them as attractive molecules for disease prognosis and/or progression [11]. Circulating miRNAs have been demonstrated as important biomarkers in many diseases, as diabetes type II [25] metabolic syndrome [26] Alzheimer’s, hypertension, osteoporosis, Parkinson’s disease, and cancer [12]. Recently, a limited number of studies highlighted the role of circulating miRNAs as potential biomarkers for assessment of prognosis and/or progression in osteoarthritis [23, 27, 28]*.* However, a systematic analysis of serum miRNA profiling in osteoarthritis has not yet been performed.

In the present study, we performed miRNA profiling using high-density miRNA-arrays in serum of OA patients and in combination with bioinformatics analysis evaluated the utility of selected differentially expressed miRNAs as potential OA biomarkers.

## Methods

### Patient samples

Serum samples were collected from 12 patients with primary OA (9 females, 3 males, ages 69.83 ± 4.83 years) undergoing total knee replacement surgery at the Orthopaedics Department of the University Hospital of Larissa. Radiographs were obtained before surgery and were graded using the Kellgren-Lawrence system according to the following criteria: grade 1 (doubtful narrowing of joint space and possible osteophytes), grade 2 (definite osteophytes and possible narrowing of joint space), grade 3 (moderate multiple osteophytes, definite narrowing of joint space and some sclerosis, and possible deformity of bone ends), and grade 4 (large osteophytes, marked narrowing of joint space, severe sclerosis, and definite deformity of bone ends). All OA patients had a Kellgren-Lawrence grade ≥ 3. The assessment of the radiographs by two independent expert observers was blinded. Patients with rheumatoid arthritis and other autoimmune disease as well as chondrodysplasias, infection-induced OA, and post-traumatic OA were excluded from the study. As controls, serum samples were obtained from 12 healthy individuals (6 females, 6 males, ages 64,25 ± 5,04 years) undergoing knee fracture repair surgery, with no history of joint disease, who did not show clinical manifestations compatible with OA when specifically explored by radiography. The clinical characteristics of OA patients and the control cohort are shown in Table 1 **.** All individuals who participated in the study were of Greek origin. The study protocol conformed to the ethical guidelines of the 1975 Declaration of Helsinki as reflected in a priori approval by the local ethical committee of the University Hospital of Larissa.

**Table 1:** Clinical characteristics of OA patients and healthy controls

Sample	Group	Sex	Age	BMI	K-L score
01OA	OA	F	68	29	4
02OA	OA	M	69	29	4
03OA	OA	F	64	33.3	4
04OA	OA	F	77	33	3
05OA	OA	F	70	28	3
06OA	OA	M	71	28.5	4
07OA	OA	M	63	34.59	3
08OA	OA	F	66	27.6	4
09OA	OA	F	77	28	3
10OA	OA	F	66	32.4	4
11OA	OA	F	76	30.5	4
12OA	OA	F	71	39.1	4
1A	Control	F	65	23.79	0
2A	Control	M	75	23.5	0
3A	Control	F	63	22	0
4A	Control	M	68	29.74	0
5A	Control	M	66	29.38	0
6A	Control	F	60	23	0
7A	Control	F	68	28	0
8A	Control	M	64	29	0
9A	Control	M	63	27	0
10A	Control	F	54	30	0
11A	Control	F	63	21.6	0
12A	Control	F	62	20.2	0

Articular cartilage samples (*n* = 12) were collected from femoral condyles and tibial plateaus of the same OA patients undergoing total knee replacement surgery. Normal articular cartilage was obtained from small free cartilaginous fragments of 7 out of the 12 healthy individuals undergoing knee fracture repair surgery. Signed informed consent was obtained from all OA and healthy individuals prior to surgery and/or blood drawing.

### RNA extraction and microRNA expression profiling by microarray in serum of OA patients and healthy individuals

Total RNA was extracted from serum samples using the RiboEXTMLS kit (GeneAll, Seoul, Korea). RNA quantity and quality were evaluated using a spectrophotometer (NanoDrop® ND-1000 UV-Vis, Nanogen Inc.). Total RNA samples were spiked using the microRNA Spike-In Kit (Agilent Technologies Inc., Santa Clara, CA, USA) to assess the labeling and hybridization efficiencies. Labeling and hybridization were performed using the miRNA complete Labeling and Hybridization Kit (Agilent Technologies Inc., Santa Clara, CA, USA) according to manufacturer’s instructions. Samples were hybridized to the SurePrint G3 Human miRNA, 8X60K platform (miRBase release 21.0, Agilent Technologies Inc., Santa Clara, CA, USA) containing probes for the detection of 2549 human miRNAs. Images were scanned using Agilent Microarray Scanner (G2565CA) controlled by Agilent Scan Control 7.0 software. The signal after background subtraction was exported directly into Agilent Feature Extraction Software version 4.0.1.21 (Agilent Technologies Inc., Santa Clara, CA, USA). Normalization was performed using quantile algorithm. MicroRNAs were considered as differentially expressed (DE) if they obtained a *p* value < 0.05 and a false discovery rate (FDR) ≤ 0.05**.**

### ROC analysis

Receiver Operating Characteristic curves (ROC) analysis was performed using the MATLAB® simulation environment and was used to calculate the area under the curve (AUC) value along with standard error and 95% confidence intervals. ROC curves were considered significant with AUC value > 0.8 and a *p* value < 0.05.

### Quantitative real-time polymerase chain reaction (qRT-PCR) validation of selected miRNAs in serum of OA and healthy individuals

The expression levels of 7 selected miRNAs screened with miRNA microarrays, as mature hsa-miR-33b-3p, hsa-miR-4284, hsa-miR-663a, hsa-miR-150-5p, hsa-miR-1233-3p, hsa-miR-140-3p, and hsa-miR-671-3p, were evaluated in serum samples from 12 OA patients and 12 healthy controls. More specifically, reverse transcription was carried out with 100 ng of total RNA using MiScript II Reverse Transcription Kit (QIAGEN Inc., Valencia, CA, USA). The relative quantification of selected differentially expressed miRNAs was performed by qRT-PCR reaction with the miScript SYBR® Green PCR Kit (QIAGEN Inc., Valencia, CA, USA) and MiScript Primer Assays (QIAGEN Inc., Valencia, CA, USA), using an ABI 7300 Real-Time PCR System (Applied Biosystems, Foster City, CA, USA). Reactions were performed in duplicate. The relative expression levels were calculated using the 2^−ΔΔCT^ method. Normalized gene expression values for each gene were generated based on cycle threshold (CT) values for each of the genes. Hsa-miR-25-1 was used as endogenous control, as it exhibited minimum variance between the controls and patients in miRNA microarray analysis. Also, its stability was confirmed using the NormFinder software [29].

### Primary cultures of OA and normal and articular chondrocytes

Articular cartilage was dissected and subjected to digestion with 1 mg/ml pronase (Roche Applied Science, Mannheim, Germany) for 30 min and then the sample was centrifuged and the pellet was subjected to digestion with 1 mg/ml collagenase P (Roche Applied Science, Mannheim, Germany) for 3 h at 37 °C. Chondrocytes were counted and checked for viability using trypan blue staining. More than 95% of the cells were viable after isolation. Chondrocytes were cultured with Dulbecco’s modified Eagles medium/Ham’s F-12 (DMEM/F-12) (GIBCO, BRL, UK) plus 5% fetal bovine serum (FBS, GIBCO, BRL, UK) and 100 U/ml penicillin-streptomycin, and were incubated at 37 °C under a humidified 5% CO_2_ atmosphere until reaching confluence. Chondrocytes were kept in culture for one passage, while types I and II collagen ratios were evaluated in all samples to exclude dedifferentiation events.

### RNA extraction and quantification of miRNA expression from OA and healthy articular cartilage

Total cellular RNA was extracted from cultured chondrocytes using Trizol reagent (Invitrogen, Life Technologies, Paisley, UK). RNA quantity and quality was evaluated using a spectrophotometer (NanoDrop® ND-1000 UV-Vis, Nanogen Inc.). Reverse transcription was conducted using the SuperScript III Reverse Transcriptase kit (Invitrogen/Thermo Fisher Scientific Inc., USA), using primers specific for hsa-miR-33b-3p, has-miR-4284, hsa-miR-663a, hsa-miR-150-5p, hsa-miR-1233-3p, hsa-miR-140-3p, hsa-miR-671-3p, and U6 small nuclear RNA (RNU6B) stem-loop RT primer to generate the cDNA as previously described [30]. MiRNA expression was verified by qRT-PCR. Reactions were done in duplicate. The reactions were performed using Power SYBR Green PCR Master Mix (Applied Biosystems) and specific primers (forward and reverse) for each miRNA. For the quantification of the relative expression of each miRNA, the threshold cycle (Ct) values were normalized against the endogenous reference U6. The 2^−ΔΔCT^ method was used [30].

### Statistical analyses and data analyses

Multiparameter analyses were performed with the MATLAB ® simulation environment (The Mathworks Inc., Natick, MA, USA). In particular, data pre-processing was performed in Microsoft Excel ® and data processing was performed within the Matlab® v.7.6.0 (The MathWorks Inc. Natick, MA, USA) computing environment, using the Bioinformatics Toolbox. For background correction, the well-performing multiplicative background correction (MBC) approach was followed [31]. In terms of filtering, as low signal intensity measurements are less reliable in terms of the impact of noise on them, than high gene expression measurements, an intensity-dependent filtering [32, 33] with an absolute threshold value of 10 was used, in order to exclude low quality features. A threshold of 1 was set as a cut-off value, meaning that spot intensity should be at least the same as that of the background. The quantile normalization method was used for further processing [34]. In order to identify potentially differentially expressed (DE) genes between samples and among genes of the same experiment a two-tailed Student’s *t* test was used to test the mean differences between the two groups. Genes that received a *p* < 0.05 were considered as DE. Further on, the false discovery rate (FDR has been calculated as described previously [35–37]. There was a FDR of 0.007% for *p* < 0.05.

Continuous variables are expressed as median ± standard deviation unless indicated differently. k-means and hierarchical clustering (HCL) analyses of microRNA expression were performed using all differentially expressed (DE) miRNAs with Euclidian distance.

RNA target prediction for selected miRNAs was performed with TargetScan v. 7.0 [38] and DIANA Tools, a collection of web-tools for miRNA functional annotation analysis [39]. Functional annotation was performed with the Webgestalt web-tool [40] and DIANA tools. Furthermore, KEGG pathway enrichment analysis was conducted for the target genes using the Database for Annotation, Visualization and Integrated Discovery (DAVID) online tools [41] with the cut-off criterion of *p* < 0.05.

Venn diagrams were generated with the Venn Diagram Calculator from the Bioinformatics, Evolutionary Genomics Group of the University of Gent.

## Results

### MicroRNA microarray profiling in serum of OA patients and healthy individuals

**Fig. 1:**
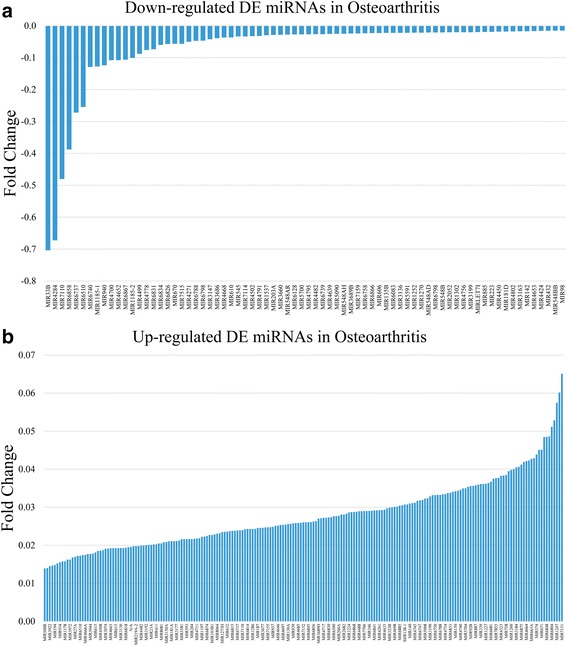
**a** Graphical representation of the *downregulated* differentially expressed (DE) miRNAs between OA patients and healthy individuals. Fold change has been estimated by calculating the *log*_*2*_ of the OA over control ratio. **b** Graphical representation of the *upregulated* differentially expressed (DE) miRNAs between OA patients and healthy individuals. Fold change has been estimated by calculating the *log*_*2*_ of the OA over control ratio

Among the 2549 miRNAs tested using the Agilent 8 × 60 K miRNA-array platform, a total of 279 miRNAs were found to be differentially expressed (205 upregulated and 74 downregulated) (*FDR* < 0.008, *p < 0.05*) in the serum of OA patients compared to healthy individuals (Fig. 1a, b **).**

Classification methods were used in order to detect patterns in gene expression. Unsupervised two-way hierarchical clustering (HCL) with Euclidian distance and k-means analyses could not discriminate accurately between OA and normal serum samples (data not shown).

### Diagnostic value of miRNAs in serum

Receiver Operating Characteristic (ROC) curves were established to evaluate the diagnostic value of deregulated miRNAs in discriminating between OA patients and healthy individuals. ROC analysis revealed that 77 miRNAs had an area under the curve (AUC) > 0.8 at a *p* < 0.05 significance level, that could thus separate OA from control samples. ROC analysis results are summarized in Additional file 1: Table S1. Among the 77 miRNAs, 7 miRNAs, hsa-miR-33b-3p, hsa-miR-4284, hsa-miR-663a, hsa-miR-150-5p, hsa-miR-1233-3p, hsa-miR-671-3p, and hsa-miR-140-3p, were selected for further validation based on the fact that they were in the top 20 up- or downregulated miRNAs as found in microarray microRNA expression profiling presented in Fig. 1a, b. More specifically, miR-33b-3p and miR-4284 were among the top upregulated, and miR-663a, miR150-5p, miR-1233-3p, miR-671-3p, and miR-140-3p were downregulated.

**Fig. 2:**
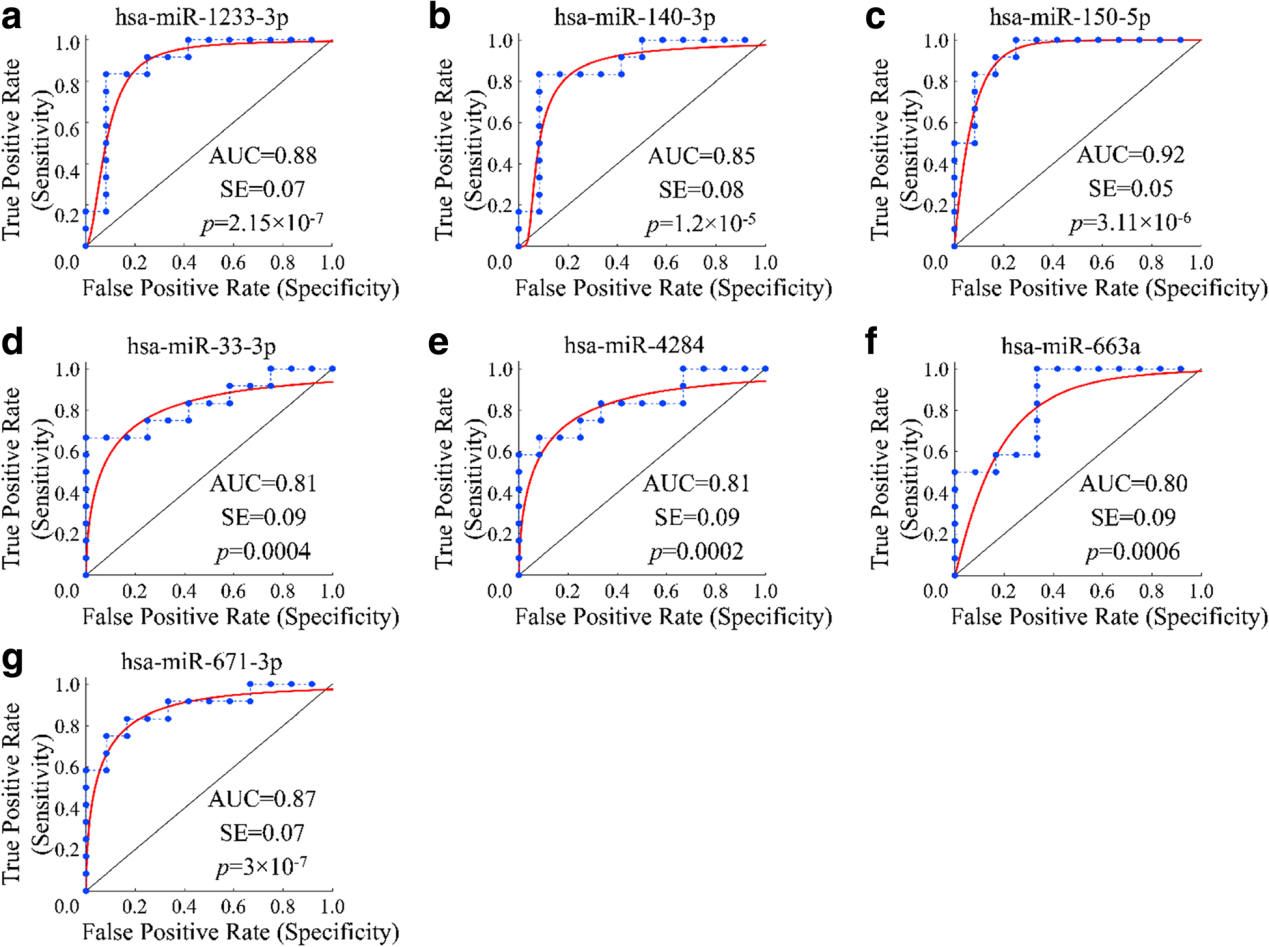
ROC analysis of miRNAs separating OA from control samples. Seven miRNAs were selected for further analysis and verification. Hsa-miR-1233-3p (**a**), hsa-miR-140-3p (**b**), hsa-miR-150-5p (**c**), hsa-miR-33-3p (**d**), hsa-miR-4284 (**e**), hsa-miR-663a (**f**), and hsa-miR-671-3p (**g**) manifested an area under the curve (AUC) value > 0.8 (AUC > 0.8) and *p* < 0.01

ROC curve analysis of the seven selected miRNAs is presented in Fig. 2 **.**

### Validation of selected miRNAs expression in serum of OA and healthy individuals by qRT-PCR

**Fig. 3:**
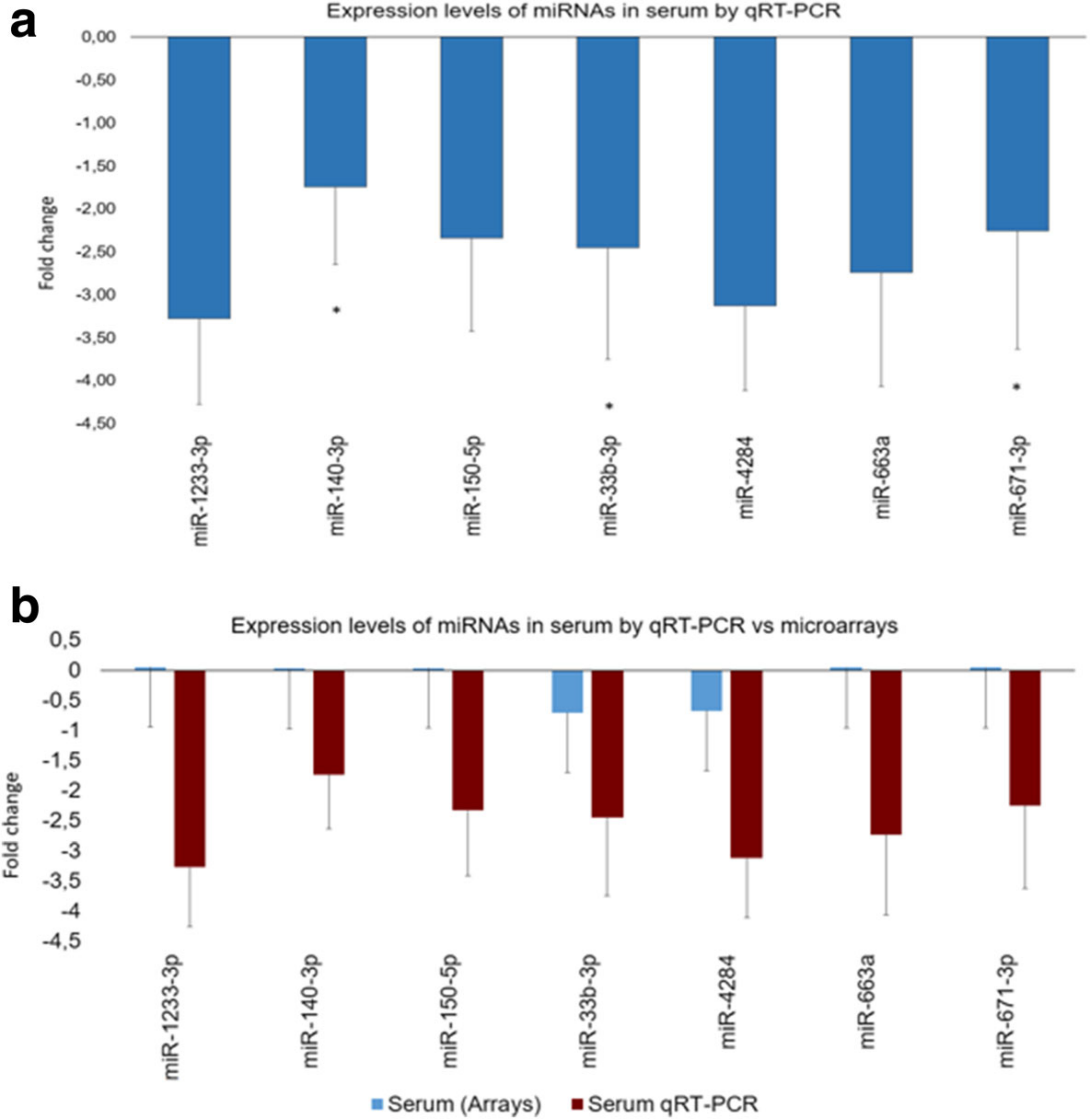
**a** Diagram of relative miRNA expression levels by qRT-PCR in the serum of healthy individuals (*n* = 12) and OA patients (*n* = 12). MiR-25-1 was used for normalization of the real-time PCR data. *p < 0.05 as measured using an unpaired Students’s *t* test. Hsa-miR-140-3p, hsa-miR-671-3p, and hsa-miR-33b-3p were significantly downregulated in serum samples of OA patients compared to healthy individuals. **b** Comparative diagram of miRNA expression levels between miRNA microarray analysis and as validated by qRT-PCR in serum samples of OA patients (*n* = 12) and healthy individuals (*n* = 12). The expression levels of hsa-miR-33b-3p and hsa-miR-4284 coincided between the two methodologies, while hsa-miR-1233-3p, hsa-miR-140-3p, hsa-miR-150-5p, hsa-miR-663a, and hsa-miR-671-3p were marginally overexpressed in microarray analysis and appeared to be down-regulated with qRT-PCR

We verified by qRT-PCR the expression levels of the seven selected miRNAs (hsa-miR-33b-3p, hsa-miR-4284, hsa-miR-663a, hsa-miR-150-5p, hsa-miR-1233-3p, hsa-miR-140-3p, and hsa-miR-671-3p) in all serum samples. Among these seven miRNAs, 3 miRNAs, hsa-miR-33b-3p, hsa-miR-671-3p, and hsa-miR-140-3p, were found significantly downregulated in OA serum samples compared to controls (*p* < 0.05) **(**Fig. 3a **)**.

Moreover, we found that hsa-miR-33b-3p and hsa-miR-4284 expression levels coincided between microarray analysis and qRT-PCR, exhibiting decreased expression in serum of OA samples compared to controls. Hsa-miR-663a, hsa-miR-150-5p, hsa-miR-1233-3p, hsa-miR-140-3p, and hsa-miR-671-3p, which were marginally over-expressed in microarray analysis, appeared to be downregulated with qRT-PCR. This discrepancy might be due to technical limitations of the microarray methodology (Fig. 3b **).**

### Pathway analysis and target gene prediction

Pathway enrichment analysis of the 77 miRNAs that were found significant in ROC analysis revealed that these DE miRNAs are potentially involved in pathways associated with OA pathogenesis, as among others, thyroid hormone, FoxO, mTOR, sphingolipoid, MAPK, PI3K-Akt, Wnt, ErbB, estrogen and TGF-beta signaling pathways, ECM-receptor interaction, and fatty acid biosynthesis. Annotated pathways are summarized in Table 2.

**Table 2:** Summary of 77 miRNAs that were found significant in ROC analysis in common annotated pathways

KEGG pathway	*p* value	#genes	#miRNAs
Proteoglycans in cancer	3.10E-11	156	64
Morphine addiction	1.07E-05	68	54
Circadian entrainment	1.07E-05	78	55
Adherens junction	3.37E-05	62	57
Phosphatidylinositol signaling system	6.91E-05	61	55
Axon guidance	6.91E-05	100	56
Glutamatergic synapse	6.91E-05	92	56
Thyroid hormone signaling pathway	0.000101627	90	62
Ras signaling pathway	0.000108175	167	66
Fatty acid biosynthesis	0.000111286	9	17
Retrograde endocannabinoid signaling	0.000113352	78	52
Rap1 signaling pathway	0.000113352	157	64
Dopaminergic synapse	0.000186561	101	57
Pathways in cancer	0.000227243	284	69
Hippo signaling pathway	0.000362414	111	62
N-Glycan biosynthesis	0.000416091	35	41
Inflammatory mediator regulation of TRP channels	0.000430502	74	48
GABAergic synapse	0.000725271	68	58
Long-term depression	0.001178836	48	46
Serotonergic synapse	0.001245646	81	51
TGF-beta signaling pathway	0.001498476	58	54
Pancreatic cancer	0.001536223	53	49
FoxO signaling pathway	0.001905724	98	59
ErbB signaling pathway	0.002223208	66	51
MAPK signaling pathway	0.002252012	183	66
Signaling pathways regulating pluripotency of stem cells	0.002265893	102	61
Wnt signaling pathway	0.002265893	104	63
Melanoma	0.002526033	56	49
Long-term potentiation	0.002526033	54	52
Nicotine addiction	0.004752071	29	43
Prion diseases	0.006877062	20	36
Circadian rhythm	0.006877062	26	41
PI3K-Akt signaling pathway	0.006877062	235	70
Prostate cancer	0.006944825	67	57
Neurotrophin signaling pathway	0.008178726	88	58
Oxytocin signaling pathway	0.008178726	111	62
mTOR signaling pathway	0.00860707	48	54
Endocrine and other factor-regulated calcium reabsorption	0.009616617	34	47
Renal cell carcinoma	0.009616617	52	47
Platelet activation	0.010946918	94	55
Adrenergic signaling in cardiomyocytes	0.010946918	102	60
Endocytosis	0.010946918	144	63
Non-small cell lung cancer	0.011911365	43	47
Cholinergic synapse	0.012031519	79	56
Glioma	0.012204133	47	48
cGMP-PKG signaling pathway	0.01268481	117	67
Endometrial cancer	0.01424854	40	54
Estrogen signaling pathway	0.01424854	70	55
Choline metabolism in cancer	0.01424854	73	62
Chronic myeloid leukemia	0.01491545	54	52
Calcium signaling pathway	0.01491545	126	59
GnRH signaling pathway	0.015627113	68	51
Gap junction	0.015668053	62	51
Amphetamine addiction	0.016450563	49	52
T cell receptor signaling pathway	0.018715138	76	52
Glycosaminoglycan biosynthesis-heparan sulfate/heparin	0.021909011	18	29
Dorso-ventral axis formation	0.021911194	23	40
ECM-receptor interaction	0.02302011	59	49
Focal adhesion	0.023875829	143	62
Regulation of actin cytoskeleton	0.027660198	149	66
Sphingolipid signaling pathway	0.035172679	80	57
Small cell lung cancer	0.046657913	62	45
Cocaine addiction	0.046657913	36	49
Ubiquitin-mediated proteolysis	0.046657913	98	61

**Fig. 4:**
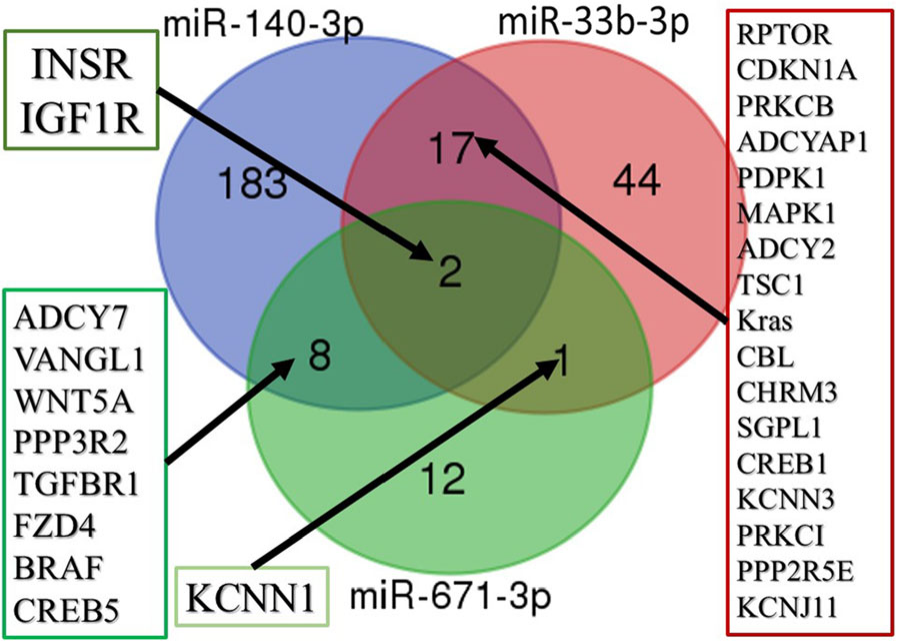
Venn diagram of common target genes between miR-140-3p, miR-33b-3p and miR-671-3p

Furthermore, by considering the qRT-PCR data, we focused on predicted target genes, by TargetScan, of the three miRNAs (hsa-miR-140-3p, hsa-miR-33b-3p, and hsa-miR-671-3p) that were found downregulated in the serum of OA patients. It appeared **(**Fig. 4 **)** that these genes are predicted targets of two or more of the three miRNAs. More specifically, 28 genes were co-targeted by at least 2 miRNAs. Among these, *INSR* and *IGF1R* were targeted by all three miRNAs, while *ADCY7, VANGL1, WNT5A, PPP3R2, TGFBR1, FZD4, BRAF,* and *CREB5* were co-targeted by hsa-miR-140-3p, and hsa-miR-671-3p, *RPTOR, CDKN1A, PRKCB, ADCYA1, PDPK1, MAPK1, ADCY2, TCS1, Kras, CBL, CHRM3, SGL1, CREB1, KCNN3, PRKC1, PPP2R5E,* and *KCNJ11* were co-targeted by hsa-miR-33b-3p, and hsa-miR-140-3p and *KCNN1* were co-targeted by hsa-miR-671-3p and hsa-miR-33b-3p.

**Fig. 5:**
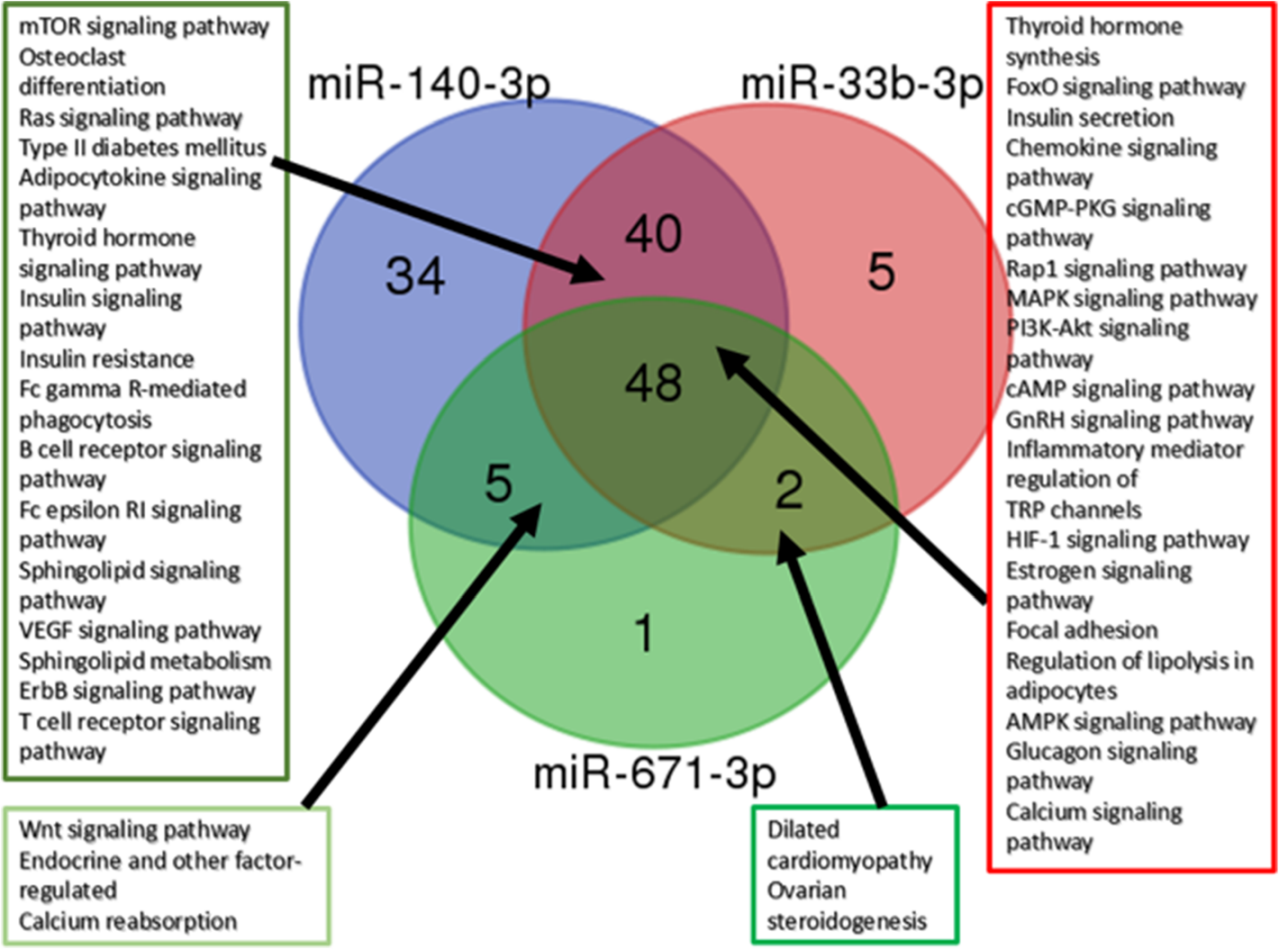
Venn diagram of common signaling pathways between miR-140-3p, miR-33b-3p and miR-671-3p

Pathway enrichment analysis (Additional file 2: Table S2) revealed that the target genes of hsa-miR-140-3p, hsa-miR-33b-3p,and hsa-miR-671-3p are potentially involved in 48 common signaling pathways, including thyroid hormone synthesis, FoxO signaling pathway, insulin secretion, chemokine signaling pathway, MAPK signaling pathway, PI3K-Akt signaling pathway, estrogen signaling pathway, regulation of lipolysis in adipocytes, glucagon signaling pathway, and calcium signaling pathway, whereas 40 common pathways were predicted common between miR-140-3p and miR-33b-3p, such as mTOR signaling pathway, osteoclast differentiation, Ras signaling pathway, type II diabetes mellitus, adipocytokine signaling pathway, thyroid hormone signaling pathway, insulin signaling pathway, insulin resistance, sphingolipid signaling pathway, sphingolipid metabolism, and ErbB signaling pathway. Furthemore, five pathways were predicted between miR-140-3p and miR-671-3p, including Wnt signaling pathway, endocrine and other factor-regulated, and calcium reabsorption (Fig. 5 **).**

### Disease annotation

Based on the previous list of the revealed miRNAs, we further investigated their association with known diseases. It appeared that the miRNAs we identified participate in arthritis, joint diseases, and rheumatic disease. Interestingly, our identified miRNAs included hsa-miR-140-3p, which participated in arthritis, joint diseases, and rheumatoid disease. On average, hsa-miR-140-3p was found to be downregulated in all OA samples. The results of the Disease Annotation are summarized in Table 3.

**Table 3:** Disease annotation of participation of selected miRNAs among others in arthritis, joint diseases, and rheumatic diseases

Index	UserID	Value	Gene symbol	Gene name	EntrezGene	Ensembl
Database: disease Name: Carcinoma, Small Cell ID:DB_ID:PA446661						
*C* = 278; *O* = 3; *E* = 0.21; *R* = 14.54; rawP = 0.0011; adjP = 0.0121						
1	MIR185	NA	MIR185	microRNA 185	406,961	NULL
2	MIR138-1	NA	MIR138-1	microRNA 138-1	406,929	NULL
3	MIR200A	NA	MIR200A	microRNA 200a	406,983	NULL
Database: disease Name: Lymphoma, Large-Cell, Diffuse ID:DB_ID:PA446313						
*C* = 262; *O* = 2; *E* = 0.19; *R* = 10.29; rawP = 0.0162; adjP = 0.0307						
1	MIR4488	NA	MIR4488	microRNA 4488	100,616,470	NULL
2	MIR4492	NA	MIR4492	microRNA 4492	100,616,376	NULL
Database: disease Name: Arthritis ID:DB_ID:PA443430						
*C* = 302; O = 2; *E* = 0.22; *R* = 8.93; rawP = 0.0211; adjP = 0.0307						
1	MIR140	NA	MIR140	microRNA 140	406,932	NULL
2	MIR346	NA	MIR346	microRNA 346	442,911	NULL
Database: disease Name: Neoplasms ID:DB_ID:PA445062						
*C* = 854; *O* = 3; *E* = 0.63; *R* = 4.73; rawP = 0.0251; adjP = 0.0307						
1	MIR185	NA	MIR185	microRNA 185	406,961	NULL
2	MIR138-1	NA	MIR138-1	microRNA 138-1	406,929	NULL
3	MIR200A	NA	MIR200A	microRNA 200a	406,983	NULL
Database: disease Name: Hematologic Neoplasms ID:DB_ID:PA446827						
*C* = 273; O = 2; *E* = 0.20; *R* = 9.87; rawP = 0.0175; adjP = 0.0307						
1	MIR671	NA	MIR671	microRNA 671	768,213	NULL
2	MIR758	NA	MIR758	microRNA 758	768,212	NULL
Database: disease Name: Brain Neoplasms ID:DB_ID:PA443557						
*C* = 198; O = 2; *E* = 0.15; *R* = 13.61; rawP = 0.0095; adjP = 0.0307						
1	MIR326	NA	MIR326	microRNA 326	442,900	NULL
2	MIR200A	NA	MIR200A	microRNA 200a	406,983	NULL
Database: disease Name: Joint Diseases ID:DB_ID:PA444661						
*C* = 266; O = 2; *E* = 0.20; *R* = 10.13; rawP = 0.0166; adjP = 0.0307						
1	MIR140	NA	MIR140	microRNA 140	406,932	NULL
2	MIR346	NA	MIR346	microRNA 346	442,911	NULL
Database:disease Name: Rheumatic Diseases ID:DB_ID:PA445555						
*C* = 295; O = 2; *E* = 0.22; *R* = 9.14; rawP = 0.0202; adjP = 0.0307						
1	MIR140	NA	MIR140	microRNA 140	406,932	NULL
2	MIR346	NA	MIR346	microRNA 346	442,911	NULL
Database: disease Name: Hematologic Diseases ID:DB_ID:PA444395						
*C* = 316; O = 2; *E* = 0.23; *R* = 8.53; rawP = 0.0230; adjP = 0.0307						
1	MIR671	NA	MIR671	microRNA 671	768,213	NULL
2	MIR150	NA	MIR150	microRNA 150	406,942	NULL
Database: disease Name: Carcinoma ID:DB_ID:PA443610						
*C* = 522; O = 2; *E* = 0.39; *R* = 5.16; rawP = 0.0571; adjP = 0.0628						
1	MIR138-1	NA	MIR138-1	microRNA 138-1	406,929	NULL
2	MIR200A	NA	MIR200A	microRNA 200a	406,983	NULL

### miRNA expression in OA and healthy articular cartilage samples

**Fig. 6:**
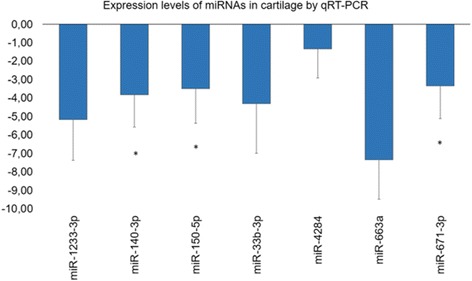
Diagram of relative miRNA expression levels by qRT-PCR in normal (*n* = 7) and osteoarthritic (OA) chondrocytes (*n* = 12). U6 was used for normalization of the real-time PCR data. All miRNAs appeared to be downregulated with respect to control samples. Fold change has been calculated as the log2 transformed ratio of the 2^−ΔΔCt^ of OA samples over control samples (asterisks depict a significant difference between the 2^−ΔΔCt^ of OA samples and 2^−ΔΔCt^ of control samples in cartilage tissues at the *p* < 0.05 level). Hsa-miR-140-3p, hsa-miR-671-3p, and hsa-miR-150p were significantly downregulated in serum samples of OA patients compared to healthy individuals

We next evaluated by qRT-PCR the expression levels of the 7 selected miRNAs (hsa-miR-33b-3p, hsa-miR-4284, hsa-miR-663a, hsa-miR-150-5p, hsa-miR-1233-3p, hsa-miR-140-3p and hsa-miR-671-3p) in 12 OA and 7 healthy articular cartilage samples. The expression levels of hsa-miR-140-3p, hsa-miR-150-5p, and hsa-miR-671-3p were significantly decreased in OA articular cartilage compared to healthy cartilage (*p* < 0.05) (Fig. 6 **).** No significant differences were observed for hsa-miR-33-3p, hsa-miR-4284, hsa-miR-663a, and hsa-miR-1233-3p expression levels between OA and healthy articular cartilage.

## Discussion

To our knowledge, this is the first study to establish a potential global serum miRNA signature in OA patients using a high-resolution microarray technology interrogating 2549 miRNAs. We identified a three-miRNA signature, including hsa-miR-140-3p, hsa-miR-671-3p, and hsa-miR-33b-3p, which was downregulated in the serum of OA patients compared to controls and in silico predicted to be involved in regulating metabolic factors.

Recent evidence points that circulating miRNAs could derive from tissue injury [42] or adipose tissue depots [43], it could thus be speculated that serum miRNAs in OA could potentially have derived from cartilage degeneration, apoptosis, or inflammation, and miRNA expression profiling in OA serum could thus constitute a disease fingerprint.

To date, limited studies have been conducted, mainly using low-density arrays and qRT-PCR for the identification of circulating miRNA expression profile in serum, plasma or blood of OA patients, with inconsistent results among different studies. More specifically, Okuhara et al., investigated the expression of hsa-miR-146a, hsa-miR-155, hsa-miR-181a, and hsa-miR-223 by qRT-PCR in peripheral blood mononuclear cells of OA patients and healthy controls and correlated their expression to disease progression [44]. In a subsequent study, the expression of 380 miRNAs using TaqMan low density arrays was evaluated in the plasma of patients with primary osteoarthritis and controls and 12 miRNAs (hsa-miR-16, hsa-miR-20b, hsa-miR-29c, hsa-miR-30b, hsa-miR-93, hsa-miR-126, hsa-miR-146a, hsa-miR-184, hsa-miR-186, hsa-miR-195, hsa-miR-345, and hsa-miR-885) were identified being over-expressed in OA patients [28]. A more recent study by Beyer et al. [27] identified let-7e in the serum of a large population-based cohort of OA patients necessitating arthroplasty as a potential predictor for severe knee or hip osteoarthritis; however, its expression was not confirmed in OA articular cartilage samples. The inability among the published studies to reveal consistent differentially expressed circulating miRNAs in OA could be attributed to differences in the study design, as selection of patients, disease status, and differences in ethnicity [45], and to different platforms and bioinformatics tools used in each study.

In the current setting among the 2549 miRNAs screened using a high-density microarray platform, we identified 279 miRNAs of which 205 upregulated and 74 downregulated, that were differentially expressed in serum between OA patients and healthy individuals. Hierarchical clustering analysis (HCA) using the miRNA signatures failed to demonstrate clear differences between OA and control serum samples. This inability of HCA might be due to the small sample size or to other technical issues, as the low dynamic range of miRNA microarrays compared to qRT-PCR to accurately identify fold-changes for miRNAs present in both high and low abundance [46].

In order to evaluate the diagnostic role of the circulating miRNAs in OA serum, we established ROC curves using a strict filtering approach. We determined 77 miRNAs that showed significant sensitivity and specificity with area AUC values > 0.8, suggesting those miRNAs as potential OA biomarkers. Many of these miRNAs have yet unknown roles that remain to be revealed. To evaluate the biological information provided by the 77 miRNAs through ROC analysis, we performed pathway enrichment analysis, which revealed that their target genes were commonly enriched in OA related pathways, such as ECM-receptor interaction, mTOR, PI3K/Akt, Wnt, TGF-β and adipocytokine signaling pathways, insulin resistance, FoxO, autophagy, and fatty acid metabolism **(**Table 2 **)**.

We, next, validated with qRT-PCR, which offers high accuracy, sensitivity, and dynamic range [46] the expression levels of 7 selected out of the 77 DE miRNAs, which were among the top 20 up- or downregulated in the microarray screening, namely, hsa-miR-33b-3p, hsa-miR-4284, hsa-miR-663a, hsa-miR-150-5p, hsa-miR-1233-3p, hsa-miR-140-3p, and hsa-miR-671-3p, in serum and in OA and healthy articular cartilage samples. We found that three miRNAs, hsa-miR-140-3p, hsa-miR-671-3p, and hsa-miR-33b-3p, were significantly downregulated in the serum of OA patients compared to controls, and in addition, hsa-miR-140-3p and hsa-miR-671-3p were also significantly downregulated in OA compared to healthy articular cartilage **(**Figs. 3a, b and 6 **)**.

Hsa-miR-140 plays an important role in cartilage homeostasis and chondrogenesis [47–49] and has been previously reported with differential expression in OA cartilage [44, 50] but not in serum. Recently, it was shown that miR-140 is required for adipogenesis [51]and that decreased levels of miR-140 were found in the plasma of patients with morbid obesity influencing *TGFbR1* expression levels [51], suggesting a new role of miR-140 in metabolic processes and obesity, main contributors in OA development. Disease Association Annotation in our study revealed that among the differentially expressed miRNAs, hsa-miR-140-3p was involved in arthritic diseases. Regarding hsa-miR-671-3p and hsa-miR-33b, there are no reports on their involvement in OA pathogenesis. Human miR-33 has two isoforms, has-miR-33a and has-miR-33b, which share the same seed sequence, have the same predicted targets, and are both regulating lipid and cholesterol metabolism [19, 52]. Our group has highlighted the role of hsa-miR-33a in regulating cholesterol transport genes [53]. Recently, miR-33b was shown to regulate adipocyte differentiation [54] and miR-33a different functions of macrophages having a role in development and progress of atherosclerosis [55]. Regarding miR-671, besides its role in cancer cell migration [56], it was recently indicated that it regulates apoptotic genes such as *caspase 8, p38*, Myc-associated factor X (*MAX*), and Ras protein-specific guanine nucleotide releasing factor *1 (RASGRF1)* in neurons of mice [56] and that it suppresses macrophage-mediated inflammation in orbital fat-derived stem cells by upregulating *IL-IRA* and *TNFRII* expressions [57, 58] highlighting its potential role in apoptosis and inflammation, both processes involved in OA.

Target gene analysis of hsa-miR-140-3p, hsa-miR-33b-3p, and hsa-miR-671-3p revealed that 28 genes were co-targeted by at least two miRNAs. Among common target-genes were insulin-like growth factor 1 receptor (*IGF1R),* insulin receptor (*InsR),* transforming growth factor beta receptor 1 (T*GFβR1)*, cAMP responsive element binding protein 5 (*CREB5),* nuclear factor kB *(NFkB),* and tumor necrosis factor a *(TNFa),* genes enriched in inflammatory and metabolic processes as revealed by pathway analysis **(**Figs. 4 and 5 **)**. An interesting finding in the in silico prediction analysis was that *InsR and IGFR1* were common targets of all three hsa-miR-140-3p, hsa-miR-33b-3p, and hsa-miR-671. Insulin is mediating its effects by binding to the specific high affinity insulin receptor (InsR), or with lower affinity to the structural- and functionally related insulin-like growth factor receptor (*IGFR*) [59]. Taking into consideration that OA is a metabolic disease [6], that insulin resistance plays a key role in the metabolic syndrome [3], and that recently a correlation was demonstrated between radiographic OA severity and insulin resistance [7], our in silico prediction analysis adds to the above evidence, suggesting a possible role of insulin resistance in OA. Along the same thought process, another interesting finding in our study was that cAMP responsive element binding protein 5 (*CREB5),* co-targeted by hsa-miR-140-3p and hsa-miR-671-3p, was significantly enriched in insulin secretion and TNF signaling pathways. CREB5 is a primary regulator of adipogenesis, that is also involved in regulating innate immune system, aging-associated inflammation and glucose homeostasis [60, 61] and under obese conditions promotes insulin resistance by activating the transcriptional repressor ATF3 and by downregulating the expression of adiponectin as well as insulin-sensitive glucose transporter 4 (*GLUT4*) [62].

All above suggest that the in silico predictions in the present study are highlighting the possible implication of circulating hsa-miR-140-3p, hsa-miR-33b-3p, and hsa-miR-671-3p as novel serum-based biomarkers for osteoarthritis prognosis and underlining their involvement in the regulation of metabolic processes that contribute to OA pathology. However, functional studies need to be conducted to verify our bioinformatics analysis and to shed light on the metabolic pathways through which the above miRNA signature leads to OA onset and development. In addition, standardized protocols for circulating miRNA isolation and analysis in larger cohorts of different ethnicities must be established before their use in clinical practice.

## Conclusions

A serum miRNA signature was established, for the first time, using high-density resolution miR-arrays in OA patients, and their deregulation was verified in articular cartilage samples. We identified a three-miRNA signature, hsa-miR-140-3p, hsa-miR-671-3p, and hsa-miR-33b-3p, in serum predicted to regulate metabolic processes, which could serve as a potential biomarker for the evaluation of OA risk and progression.

## Additional files

Additional file 1: ROC analysis of miRNA expression values for OA and control serum samples. (DOCX 18 kb)

Additional file 2: Pathway enrichment analysis for target genes of hsa-miR-33b-3p, hsa-miR-140-3p and hsa-miR-671-3p using DAVID database (*p* < 0.05). (XLSX 29 kb)

## Abbreviations

AUC: Area under the curve
*CREB5*: cAMP responsive element binding protein 5
CT: Cycle threshold
DAVID: Database for Annotation, Visualization and Integrated Discovery
DE: Differentially expressed
FBS: Fetal bovine serum
FDR: False discovery rate
*GLUT4*: Insulin-sensitive glucose transporter 4
HCA: Hierarchical clustering analysis
HCL: Hierarchical clustering
HDL: High-density lipoprotein
*IGF1R*: Insulin-like growth factor 1 receptor
*IGFR*: Insulin-like growth factor receptor
*InsR*: High affinity insulin receptor
MAX: Myc-associated factor X
miRNAs: MicroRNAs
*NFkB*: Nuclear Factor kB
OA: Osteoarthritis
qRT-PCR: Quantitative real-time PCR
*RASGRF1*: Ras protein-specific guanine nucleotide releasing factor *1*
ROC: Receiver Operating Characteristic curves
T*GFβR1*: Transforming growth factor beta receptor 1
*TNFa*: Tumor necrosis factor a
