# Supplementary material for: Serum microRNA array analysis identifies miR-140-3p, miR-33b-3p and miR-671-3p as potential osteoarthritis biomarkers involved in metabolic processes
Source: Clin Epigenetics. 2017 Dec 12;9:127. doi: 10.1186/s13148-017-0428-1 (PMC5728069; doi:10.1186/s13148-017-0428-1)
Supplement: Supplementary file 1 — ROC analysis of miRNA expression values for OA and control serum samples. (DOCX 18 kb) [file 13148_2017_428_MOESM1_ESM.docx]

**Supplementary Table 1.** ROC analysis of miRNA expression values for OA and control serum samples

| **Inv.** | **ID** | **SYMBOL** | **AUC** | **SE** | **95% CI Lower** | **95% CI Upper** |
| --- | --- | --- | --- | --- | --- | --- |
| 1 | hsa-miR-6816-5p | MIR6816 | 0.80082 | 0.09247 | 0.61959 | 0.98205 |
| 2 | hsa-miR-3660 | MIR3660 | 0.80100 | 0.09243 | 0.61983 | 0.98216 |
| 3 | hsa-miR-326 | MIR326 | 0.80245 | 0.09214 | 0.62186 | 0.98304 |
| 4 | hsa-miR-4673 | MIR4673 | 0.80275 | 0.09208 | 0.62228 | 0.98322 |
| 5 | hsa-miR-5008-3p | MIR5008 | 0.80325 | 0.09198 | 0.62297 | 0.98352 |
| 6 | hsa-miR-4778-5p | MIR4778 | 0.80450 | 0.09172 | 0.62472 | 0.98427 |
| 7 | hsa-miR-6747-3p | MIR6747 | 0.80472 | 0.09168 | 0.62503 | 0.98441 |
| 8 | hsa-miR-33b-3p | MIR33B | 0.80532 | 0.09156 | 0.62587 | 0.98477 |
| 9 | hsa-miR-2682-3p | MIR2682 | 0.80540 | 0.09154 | 0.62598 | 0.98482 |
| 10 | hsa-miR-567 | MIR567 | 0.80569 | 0.09148 | 0.62639 | 0.98499 |
| 11 | hsa-miR-3074-5p | MIR3074 | 0.80669 | 0.09128 | 0.62780 | 0.98559 |
| 12 | hsa-miR-6798-3p | MIR6798 | 0.80697 | 0.09122 | 0.62818 | 0.98575 |
| 13 | hsa-miR-6788-3p | MIR6788 | 0.80886 | 0.09083 | 0.63084 | 0.98688 |
| 14 | hsa-miR-4695-3p | MIR4695 | 0.80943 | 0.09071 | 0.63164 | 0.98722 |
| 15 | hsa-miR-6823-3p | MIR6823 | 0.81035 | 0.09052 | 0.63294 | 0.98777 |
| 16 | hsa-miR-6866-5p | MIR6866 | 0.81040 | 0.09051 | 0.63300 | 0.98779 |
| 17 | hsa-miR-4482-5p | MIR4482 | 0.81160 | 0.09026 | 0.63469 | 0.98850 |
| 18 | hsa-miR-1260b | MIR1260B | 0.81197 | 0.09018 | 0.63522 | 0.98872 |
| 19 | hsa-miR-1284 | MIR1284 | 0.81206 | 0.09016 | 0.63534 | 0.98877 |
| 20 | hsa-miR-4700-3p | MIR4700 | 0.81340 | 0.08988 | 0.63725 | 0.98956 |
| 21 | hsa-miR-4284 | MIR4284 | 0.81372 | 0.08981 | 0.63770 | 0.98974 |
| 22 | hsa-miR-6128 | MIR6128 | 0.81396 | 0.08976 | 0.63804 | 0.98988 |
| 23 | hsa-miR-670-5p | MIR670 | 0.81400 | 0.08975 | 0.63809 | 0.98991 |
| 24 | hsa-miR-7155-3p | MIR7155 | 0.81644 | 0.08923 | 0.64156 | 0.99133 |
| 25 | hsa-miR-4652-3p | MIR4652 | 0.81692 | 0.08913 | 0.64224 | 0.99160 |
| 26 | hsa-miR-548b-5p | MIR548B | 0.81700 | 0.08911 | 0.64235 | 0.99165 |
| 27 | hsa-miR-6833-3p | MIR6833 | 0.81724 | 0.08906 | 0.64269 | 0.99179 |
| 28 | hsa-miR-6840-5p | MIR6840 | 0.81921 | 0.08863 | 0.64550 | 0.99292 |
| 29 | hsa-miR-545-5p | MIR545 | 0.81980 | 0.08850 | 0.64633 | 0.99326 |
| 30 | hsa-miR-6750-3p | MIR6750 | 0.82135 | 0.08816 | 0.64856 | 0.99415 |
| 31 | hsa-miR-23a-3p | MIR23A | 0.82142 | 0.08815 | 0.64866 | 0.99419 |
| 32 | hsa-miR-200a-5p | MIR200A | 0.82189 | 0.08804 | 0.64932 | 0.99445 |
| 33 | hsa-miR-3136-5p | MIR3136 | 0.82237 | 0.08794 | 0.65002 | 0.99473 |
| 34 | hsa-miR-1224-3p | MIR1224 | 0.82263 | 0.08788 | 0.65039 | 0.99487 |
| 35 | hsa-miR-675-5p | MIR675 | 0.82284 | 0.08783 | 0.65069 | 0.99499 |
| 36 | hsa-miR-6834-3p | MIR6834 | 0.82322 | 0.08775 | 0.65123 | 0.99521 |
| 37 | hsa-miR-4711-5p | MIR4711 | 0.82339 | 0.08771 | 0.65147 | 0.99530 |
| 38 | hsa-miR-664a-3p | MIR664A | 0.82439 | 0.08749 | 0.65291 | 0.99587 |
| 39 | hsa-miR-5193 | MIR5193 | 0.82526 | 0.08730 | 0.65416 | 0.99636 |
| 40 | hsa-miR-4740-5p | MIR4740 | 0.82709 | 0.08688 | 0.65680 | 0.99738 |
| 41 | hsa-miR-6737-5p | MIR6737 | 0.82904 | 0.08644 | 0.65962 | 0.99847 |
| 42 | hsa-miR-6808-3p | MIR6808 | 0.82968 | 0.08630 | 0.66054 | 0.99882 |
| 43 | hsa-miR-4488 | MIR4488 | 0.83088 | 0.08602 | 0.66228 | 0.99948 |
| 44 | hsa-miR-4761-5p | MIR4761 | 0.83110 | 0.08597 | 0.66259 | 0.99960 |
| 45 | hsa-miR-6788-5p | MIR6788 | 0.83261 | 0.08562 | 0.66480 | 1.00000 |
| 46 | hsa-miR-4639-3p | MIR4639 | 0.83313 | 0.08550 | 0.66555 | 1.00000 |
| 47 | hsa-miR-6858-3p | MIR6858 | 0.83386 | 0.08533 | 0.66661 | 1.00000 |
| 48 | hsa-miR-1296-5p | MIR1296 | 0.83518 | 0.08503 | 0.66853 | 1.00000 |
| 49 | hsa-miR-7855-5p | MIR7855 | 0.83579 | 0.08488 | 0.66942 | 1.00000 |
| 50 | hsa-miR-1227-5p | MIR1227 | 0.83761 | 0.08445 | 0.67209 | 1.00000 |
| 51 | hsa-miR-5011-5p | MIR5011 | 0.83837 | 0.08427 | 0.67319 | 1.00000 |
| 52 | hsa-miR-548ao-5p | MIR548A | 0.83976 | 0.08394 | 0.67524 | 1.00000 |
| 53 | hsa-miR-3130-3p | MIR3130 | 0.83984 | 0.08392 | 0.67536 | 1.00000 |
| 54 | hsa-miR-1537-3p | MIR1537 | 0.84468 | 0.08275 | 0.68249 | 1.00000 |
| 55 | hsa-miR-3714 | MIR3714 | 0.84621 | 0.08237 | 0.68476 | 1.00000 |
| 56 | hsa-miR-140-3p | MIR140 | 0.84695 | 0.08219 | 0.68586 | 1.00000 |
| 57 | hsa-miR-6081 | MIR6081 | 0.84710 | 0.08215 | 0.68609 | 1.00000 |
| 58 | hsa-miR-6798-5p | MIR6798 | 0.85202 | 0.08092 | 0.69341 | 1.00000 |
| 59 | hsa-miR-346 | MIR346 | 0.85548 | 0.08003 | 0.69862 | 1.00000 |
| 60 | hsa-miR-185-3p | MIR185 | 0.85916 | 0.07908 | 0.70417 | 1.00000 |
| 61 | hsa-miR-4492 | MIR4492 | 0.86282 | 0.07810 | 0.70974 | 1.00000 |
| 62 | hsa-miR-7515 | MIR7515 | 0.86687 | 0.07701 | 0.71593 | 1.00000 |
| 63 | hsa-miR-6814-3p | MIR6814 | 0.86709 | 0.07695 | 0.71627 | 1.00000 |
| 64 | hsa-miR-758-5p | MIR758 | 0.86979 | 0.07621 | 0.72042 | 1.00000 |
| 65 | hsa-miR-3686 | MIR3686 | 0.86984 | 0.07619 | 0.72050 | 1.00000 |
| 66 | hsa-miR-4800-3p | MIR4800 | 0.86993 | 0.07617 | 0.72064 | 1.00000 |
| 67 | hsa-miR-6781-3p | MIR6781 | 0.87169 | 0.07568 | 0.72337 | 1.00000 |
| 68 | hsa-miR-671-3p | MIR671 | 0.87430 | 0.07494 | 0.72741 | 1.00000 |
| 69 | hsa-miR-1233-3p | MIR1233 | 0.87616 | 0.07441 | 0.73032 | 1.00000 |
| 70 | hsa-miR-3147 | MIR3147 | 0.88243 | 0.07259 | 0.74016 | 1.00000 |
| 71 | hsa-miR-6868-5p | MIR6868 | 0.88530 | 0.07174 | 0.74470 | 1.00000 |
| 72 | hsa-miR-138-1-3p | MIR1381 | 0.88717 | 0.07117 | 0.74768 | 1.00000 |
| 73 | hsa-miR-7151-5p | MIR7151 | 0.89624 | 0.06835 | 0.76229 | 1.00000 |
| 74 | hsa-miR-4734 | MIR4734 | 0.90554 | 0.06529 | 0.77758 | 1.00000 |
| 75 | hsa-miR-4668-5p | MIR4668 | 0.90738 | 0.06467 | 0.78064 | 1.00000 |
| 76 | hsa-miR-6875-3p | MIR6875 | 0.92001 | 0.06018 | 0.80207 | 1.00000 |
| 77 | hsa-miR-150-5p | MIR150 | 0.92200 | 0.05943 | 0.80551 | 1.00000 |

**OA**: Osteoarthritis, **AUC**: Area under the Curve, **SE**: Standard Error, **CI**: Confidence Intervals
